# Dynamic [^18^F]PI-2620 recording facilitates prediction of β-amyloid positivity and simultaneous staging of tau and neurodegeneration

**DOI:** 10.1186/s13024-026-00997-3

**Published:** 2026-09-24

**Authors:** Johannes Gnörich, Julia Kusche-Palenga, Agnes Kling, Amir Dehsarvi, Angela Bronte, Lukas Frontzkowski, Artem Zatcepin, Mirlind Zaganjori, Florian Schöberl, Sebastian N. Roemer-Cassiano, Boris-Stephan Rauchmann, Carolin Kurz, Carla Palleis, Alexander M. Bernhardt, Alexander Jäck, Sabrina Katzdobler, Maximilian Scheifele, Theresa Bauer, Gérard N. Bischof, Thilo van Eimeren, Alexander Drzezga, Jan Häckert, Robert Perneczky, Michael Rullmann, Katharina Buerger, Rudolf A. Werner, Andreas Zwergal, Johannes Levin, Peter Bartenstein, Osama Sabri, Henryk Barthel, Sophia Stöcklein, Günter Höglinger, Nicolai Franzmeier, Matthias Brendel

**Affiliations:** 1 Department of Nuclear Medicine, LMU University Hospital, LMU Medizin, Ludwig-Maximilians-Universität München, Munich, Germany; 2 German Center for Neurodegenerative Diseases (DZNE) Munich, Munich, Germany; 3 Institute for Stroke and Dementia Research, LMU University Hospital, LMU Medizin, Ludwig-Maximilians-Universität München, Munich, Germany; 4 Universidad de Navarra, Pamplona, Spain; 5 Department of Neurology, LMU University Hospital, LMU Medizin, Ludwig-Maximilians-Universität München, Munich, Germany; 6 Department of Psychiatry and Psychotherapy, LMU University Hospital, LMU Medizin, Ludwig-Maximilians-Universität München, Munich, Germany; 7 Munich Cluster for Systems Neurology (SyNergy), Munich, Germany; 8 Department of Nuclear Medicine, Faculty of Medicine, University Hospital Cologne, Cologne, Germany; 9 Institute of Neuroscience and Medicine (INM-2), Molecular Organization of the Brain, Forschungszentrum Jülich, Germany; 10 German Center for Neurodegenerative Diseases (DZNE), Bonn, Germany; 11 Department of Psychiatry, Psychotherapy and Psychosomatics, Medical Faculty, University of Augsburg, BKH Augsburg, Augsburg, Germany; 12 Sheffield Institute for Translational Neuroscience (SITraN), University of Sheffield, Sheffield, UK; 13 Ageing Epidemiology Research Unit (AGE), School of Public Health, Imperial College London, London, UK; 14 The Russell H Morgan Department of Radiology and Radiological Sciences, Division of Nuclear Medicine, Johns Hopkins School of Medicine, Baltimore, MD 21205 USA; 15 Department of Nuclear Medicine, University Hospital Leipzig, Leipzig, Germany; 16 German Center for Vertigo and Balance Disorders, DSGZ, LMU University Hospital, LMU Medizin, Ludwig-Maximilians-Universität München, Munich, Germany; 17 Department of Radiology, LMU University Hospital, LMU Medizin, Ludwig-Maximilians-Universität München, Munich, Germany; 18 The Sahlgrenska Academy, Institute of Neuroscience and Physiology, Department of Psychiatry and Neurochemistry, University of Gothenburg, Mölndal and Gothenburg, Sweden

**Keywords:** Tau-PET, Tauopathies, Staging, β-amyloid, Dynamic imaging, A/T/N

## Abstract

**Background:**

Patients with Alzheimer’s disease (AD) and clinically overlapping neurodegenerative diseases are classified molecularly using the A/T/N classification system. Apart from fluid biomarkers and structural MRI, A/T/N assessment incorporates β-amyloid-PET (A), tau-PET (T), and [^18^F]FDG-PET (N). We evaluated if dynamic features of tau-PET with [^18^F]PI-2620 allow prediction of β-amyloid positivity and simultaneous staging of tau and neurodegeneration in individual patients using a single imaging session.

**Methods:**

We studied 129 patients with tauopathies, comprising 47 patients with β-amyloid-positive 3/4-repeat tauopathy and 82 patients with β-amyloid-negative primary 4-repeat tauopathies, alongside 17 healthy controls. Participants underwent 60-minute dynamic [^18^F]PI-2620 tau-PET. Kinetic modelling simultaneously provided tracer efflux rate (K2a) as a predictor for β-amyloid status, distribution volume ratio (DVR) for regional tau burden, and relative perfusion (R1) as a marker of neurodegeneration. These parameters were validated against β-amyloid-PET, [^18^F]FDG-PET, volumetric MRI, and cerebrospinal fluid biomarkers using receiver operating characteristic and correlation analyses and visual assessments. [^18^F]PI-2620_K2a_-based prediction of β-amyloid positivtiy was additionally tested in an independent, clinically heterogeneous validation cohort of 97 individuals.

**Results:**

[^18^F]PI-2620_K2a_ differentiated tau isoform compositions despite clinically overlapping presentations and tau-PET patterns, demonstrating reduced cortical tracer clearance in 3/4-repeat compared with 4-repeat tauopathies. [^18^F]PI-2620_K2a_ remained sensitive in individuals with visually negative or low [^18^F]PI-2620_DVR_ signals and outperformed both tau burden and perfusion for predicting β-amyloid status, achieving an area under the curve of 0.99, a positive predictive value of 91.5%, and a negative predictive value of 95.1%. Performance remained robust in the independent validation cohort, with an area under the curve of 0.98, a positive predictive value of 87.9%, and a negative predictive value of 95.3%. [^18^F]PI-2620_DVR_ quantified regional tau patterns, with cortical predominance in 3/4-repeat tauopathy and subcortical involvement in 4-repeat tauopathies. [^18^F]PI-2620_R1_ exhibited strong quantitative and visual associations with established markers of neurodegeneration. Integration of K2a, DVR, and R1 in disease-specific composite regions enabled individualized three-dimensional A/T/N staging.

**Conclusions:**

Dynamic [^18^F]PI-2620 PET imaging facilitates assessment of the β-amyloid status and regional staging of tau and neurodegeneration during a single acquisition. This one-stop-shop approach may reduce radiation exposure, streamline diagnostic workflows, and facilitate personalized disease profiling.

**Graphical Abstract:**

**Figure:**
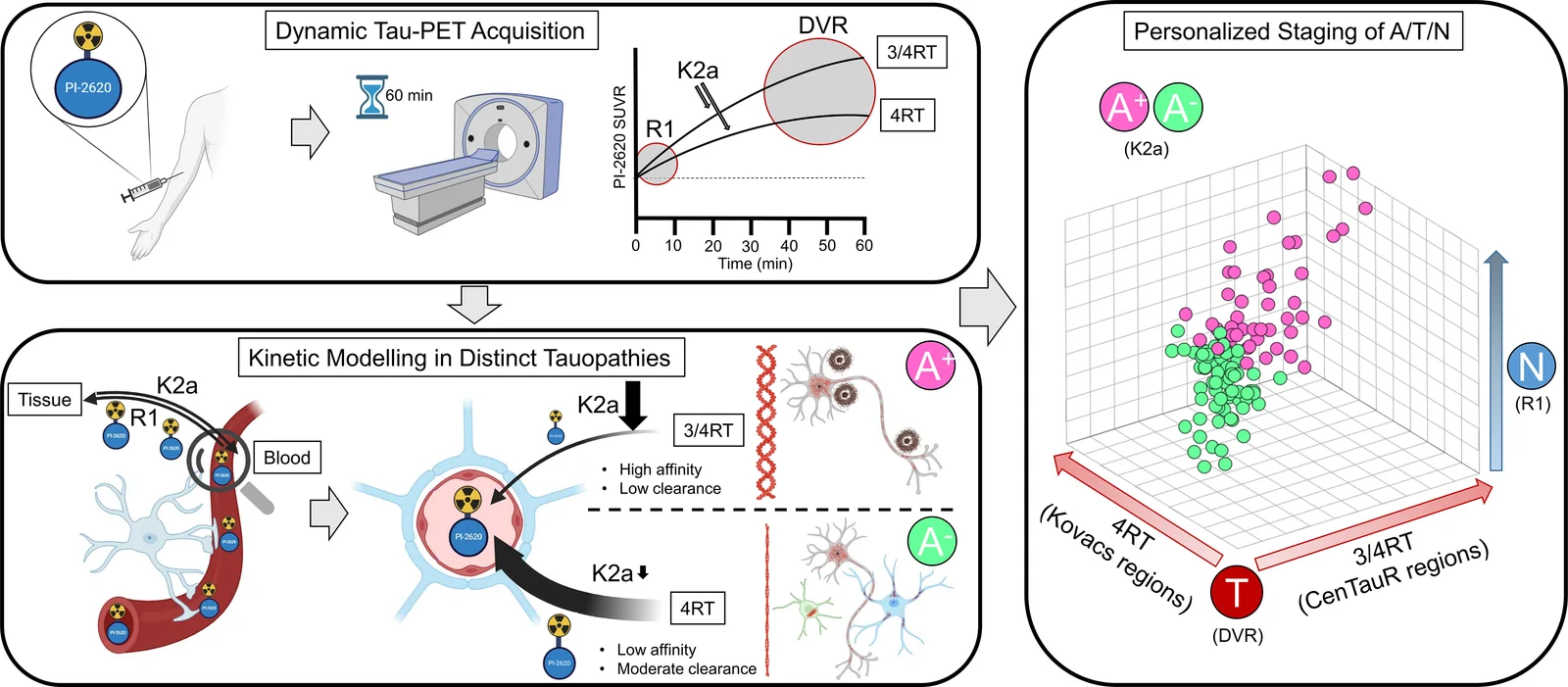

**Supplementary Information:**

The online version contains supplementary material available at https://doi.org/10.1186/s13024-026-00997-3.

## Background

Tauopathies comprise a spectrum of neurodegenerative diseases characterized by the aggregation of tau protein in the brain. These diseases are classified into primary and secondary tauopathies based on the molecular composition of tau aggregates and associated upstream protein deposits. Primary tauopathies, such as progressive supranuclear palsy (PSP) and corticobasal degeneration (CBD), are characterized by the abnormal aggregation of tau isoforms with 4-repeat (4R) binding domains, leading to distinct clinical phenotypes including atypical parkinsonian syndromes and cognitive impairments [1–3]. Clinically, the term corticobasal syndrome (CBS) is used to describe a specific presentation that is most commonly associated with CBD, but can also result from other underlying pathologies, such as PSP or Alzheimer’s disease (AD) [4]. On the other hand, AD is classically considered a secondary tauopathy, where beta-(β)-amyloid plaque deposition precedes the development of cortical 3/4R-tau aggregates [5, 6].

Imaging and fluid biomarkers are less advanced for in vivo classification of primary 4R-tauopathies (4RT), limiting our mechanistic understanding and impeding treatment development. Furthermore, the differentiation between tauopathies can be challenging due to significant clinical and pathological overlap with atypical forms of AD, often exhibiting tau pathology patterns that can mimic those seen in primary tauopathies. This overlap complicates diagnosis, particularly in the early stages of disease progression, and may lead to misclassification without comprehensive biomarker assessment.

The emergence of the second-generation tau-PET tracer [^18^F]PI-2620 holds significant promise for in vivo detection of tauopathies. Unlike first-generation tau-PET ligands, [^18^F]PI-2620 exhibits reduced off-target binding, making it a more specific tool for detecting tau pathology [7]. Its high affinity for both 3R and 4R aggregated tau isoforms, as demonstrated in vitro and in vivo studies, positions it as a valuable biomarker capable of discerning between various tauopathies [8–10]. While AD-tau filaments exhibit favourable energetic and kinetic properties for [^18^F]PI-2620 binding, interactions with CBD- and PSP-tau filaments are kinetically weaker [11]. This was also demonstrated by dynamic PET parameters derived from non-invasive reference tissue modelling, which revealed faster tracer clearance in 4RT, suggesting less stable binding compared to 3/4R-tauopathies. Notably, the parameter K2a, representing tracer efflux from the tissue, indicated potential in differentiating between distinct tau isoform compositions [12] and may therefore also serve as an indirect marker for β-amyloid pathology. Given the faster tracer washout in 4RT, earlier imaging times (i.e., 20–40 min p.i.) provide higher quantitative and visual sensitivity for detection of patients with PSP and CBS, compared to later imaging times [13, 14]. Furthermore, [^18^F]PI-2620 holds promise as a surrogate biomarker for neuronal injury in tauopathies, as evidenced by its ability to demonstrate decreased early-phase [^18^F]PI-2620 perfusion [15, 16]. The integration of perfusion and tau biomarkers enhanced biomarker-guided stratification of tauopathies within diagnostic algorithms and demonstrated improved discrimination, though it has yet to be prospectively validated [10].

These recent advancements in biomarker development, particularly in AD, have revolutionized disease diagnosis and monitoring, facilitating the establishment of diagnostic workflows and biomarker-based staging systems, as initially proposed by Jack et al. [17]. The A/T/N classification scheme offers a comprehensive framework for characterizing the underlying pathology. This pivotal scheme stratifies neurodegenerative diseases based on the presence of β-amyloid (A), tau (T), and neurodegeneration (N) biomarkers, as measured by imaging techniques and fluid biomarker assessments. Imaging by β-amyloid-PET (A), tau-PET (T), and [^18^F]FDG-PET for (N) can provide not only binary classification, but also regional staging of A/T/N biomarker abnormality. However, this approach requires multiple imaging sessions, posing an added burden to patients and the healthcare system. For this reason, there is still an unmet need to develop a straightforward solution that enables a ‘one-stop-shop’ approach for classifying patients based on A/T/N criteria.

**Fig. 1:**
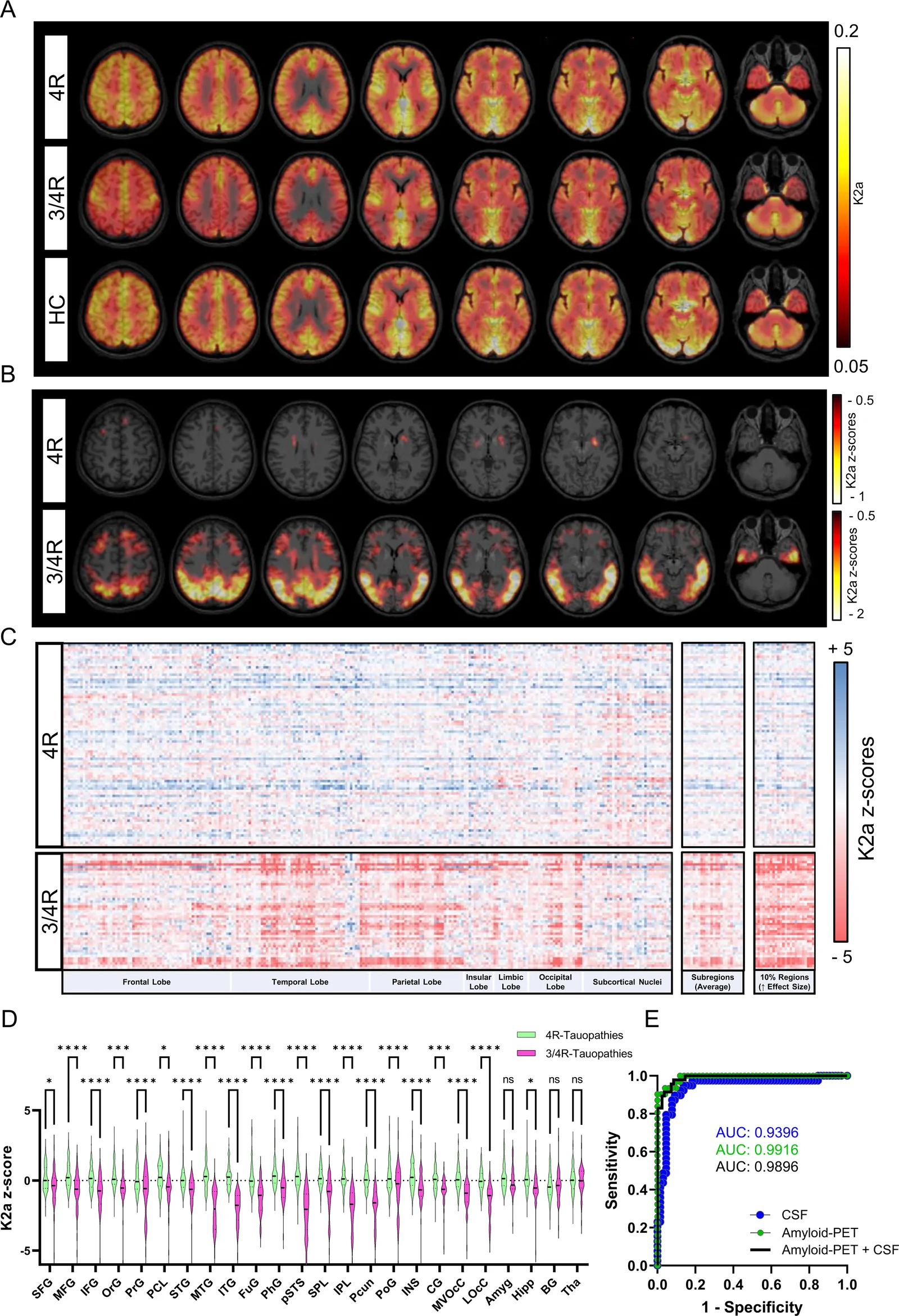
K2a assessment as an index of tracer clearance in tauopathies. (**A-B**) Average [^18^F]PI-2620 K2a and K2a z-score images of 4RT (*n* = 82), 3/4RT (*n* = 47) in relation to healthy controls (*n* = 17) (HC), illustrated by axial slice overlays on a standard MRI template in MNI space. (**C**) Heatmaps on the left show K2a z-scores of all Brainnetome atlas regions among all individuals. Heatmaps on the right demonstrate the upper 10% of discriminating regions determined by effect size analysis between both tauopathy cohorts. Intermediate heatmaps illustrate the composite Brainnetome atlas subregions, which are subsequently shown in the ANOVA analysis of panel (**D**). Significance levels are indicated as: *p < 0.0332, **p < 0.0021, ***p < 0.0002, ****p < 0.0001. (**E**) ROC curves represent the results of a logistic regression analysis using the averaged K2a of the top 10% discriminating regions against CSF (blue dots), β-amyloid-PET (green dots), and both combined (black line) to predict β-amyloid positivity in all participants. Abbreviations: SFG, superior frontal gyrus; MFG, middle frontal gyrus; IFG, inferior frontal gyrus; OrG, orbital gyrus; PrG, precentral gyrus; PCL, paracentral lobule; STG, superior temporal gyrus; MTG, middle temporal gyrus; ITG, inferior temporal gyrus; FuG, fusiform gyrus; PhG, parahippocampal gyrus; pSTS, posterior superior temporal sulcus; SPL, superior parietal lobule; IPL, inferior parietal lobule; Pcun, precuneus; PoG, postcentral gyrus; INS, insular gyrus; CG, cingulate gyrus; MVOcC, medioventral occipital cortex; LOcC, lateral occipital cortex; Amyg, amygdala; Hipp, hippocampus; BG, basal ganglia; Tha, thalamus

In this study, we assessed the value of dynamic [^18^F]PI-2620 tau-PET imaging by utilization of the (i) kinetics of radiotracer distribution as β-amyloid predictor (A), (ii) late phase binding to tau (T), and (iii) perfusion phase as a neurodegeneration surrogate (N) (Fig. 1). Our approach extends beyond traditional AD frameworks by applying the A/T/N system also to 4RT. This focus on 4RT addresses a critical gap in personalized neurodegenerative diagnostics, as these disorders often present distinct biomarker profiles compared to AD. Besides the K2a efflux rate parameter, we also extracted relative perfusion (R1) values as an indicator of tracer delivery relative to the reference region, as well as the distribution volume ratio (DVR), which reflects the relative binding potential.

Thus, we evaluated staging by all three A/T/N biomarkers in individual patients within a single dynamic imaging session. We validated our results against gold standard assessments, which involved integrating multimodal PET imaging (β-amyloid & [^18^F]FDG), volumetric cranial magnetic resonance imaging (cMRI), and cerebrospinal fluid (CSF) biomarkers (p-tau-181, t-tau & Aβ_42/40_). Finally, we developed a 3-dimensional quantitative staging index to determine personalized profiles of A/T/N-based disease severity.

## Methods

### Cohort and study design

All subjects were recruited and scanned at the Ludwig-Maximilians-University of Munich (LMU), Department of Nuclear Medicine between 2018 and 2024. Patients were diagnosed to belong to the AD continuum (total *n* = 47; ⌀75 ± 9 years, 62% female) as 3/4RT or PSP/CBS (*n* = 82; ⌀74 ± 7 years, 43% female) as 4RT according to current diagnostic criteria [3, 18, 19] and compared to healthy controls (HC) (*n* = 17; ⌀70 ± 10 years, 53% female). Patients with AD were required to meet criteria for typical or atypical AD with MCI or dementia according to the diagnostic criteria of the National Institute on Aging and Alzheimer’s Association and were only included as AD if CSF and/or PET biomarkers of β-amyloid pathology were positive [20]. Threshold for Aβ positivity was set to an Aβ42/40 ratio < 5.5% in CSF according to standardized laboratory diagnostics at LMU and/or a positive β-amyloid-PET visual read [21]. Patients with probable or possible PSP and CBS were assessed in accordance with the current diagnostic criteria, with a particular emphasis on close monitoring of disease progression during clinical follow-up (31 ± 27 months) [3, 18]. In patients with PSP, disease severity was assessed using the PSP Rating Scale, while cognitive impairment severity was evaluated using the Montreal Cognitive Assessment (MoCA). Disease duration was defined as the interval between the onset of symptoms and the date of tau-PET imaging. An independent validation cohort was defined to assess the generalizability of K2a-based prediction of β-amyloid status. This cohort comprised *n* = 97 individuals with a clinically heterogeneous spectrum of neurodegenerative syndromes, including phenotypes of PSP, CBS, and FTD (Supplementary Table 1). Diagnostic classification followed the same criteria as described above. β-amyloid status was determined using the same CSF and/or PET-based criteria. Analyses were performed independently from the main cohort without re-optimization of model parameters; regions and thresholds derived from the main cohort were applied unchanged. Demographic and biomarker data were limited to variables required for the primary validation endpoint. All participants (or their legal representatives) provided written consent for PET imaging. The study protocol and PET data analyses were approved by the local ethics committee (LMU Munich, application numbers 17–569 and 19–022). The study was carried out according to the principles of the Helsinki Declaration. A detailed overview of the study concept, analytical workflow, and application of kinetic PET parameters for individualized A/T/N staging is illustrated in the Graphical Abstract.

### CSF and plasma analyses

A subset of the participants (3/4RT, *n* = 41; 4RT, *n* = 63) underwent lumbar puncture at their visit at LMU University Hospital, Munich and their CSF levels were analyzed with ELISA Innotest Kit (Fujirebio Europe N.V., Belgium) at the MVZ laboratory PD Dr. Volkmann & Kollegen GbR in Karlsruhe, Germany. The CSF biomarkers included Aβ_42_, Aβ_40_, Aβ ratio, t-tau, and p-tau-181. The respective normal cut-off values were for Aβ_42/40_ ratio > 5.5%, t-tau < 445 pg/ml, and for p-tau-181 < 61 pg/ml, according to standardized laboratory diagnostics at the partnering laboratory [10, 22, 23]. In a small subset of participants (3/4RT: *n* = 11; 4RT: *n* = 48), plasma neurofilament light chain (NfL) levels were additionally assessed through blood analysis.

### Radiosynthesis

Radiosynthesis of [^18^F]PI-2620 was achieved by nucleophilic substitution on a tert-Butyloxycarbonyl-(BOC)-protected nitro precursor using an automated synthesis module (IBA, Synthera). The protecting group was cleaved under the radiolabelling conditions. The product was purified by semipreparative high-performance liquid chromatography (HPLC). Radiochemical purity was 99%. Non-decay corrected yields were about 35% with a molar activity of 8∙10^6^ GBq/mmol at the end of synthesis. Radiosynthesis of Flutemetamol was performed as described previously [24, 25]. Florbetaben was purchased commercially from LIFE Molecular Imaging GmbH and [^18^F]FDG from Advanced Accelerator Applications (AAA).

### Tau-PET acquisition and preprocessing

All patients were scanned at the Department of Nuclear Medicine, LMU Munich, with a Biograph 64 or a Siemens mCT PET/CT scanner (both Siemens, Erlangen, Germany). The dynamic brain PET data were acquired in 3-dimensional list-mode over 60 min and reconstructed into a 336 × 336 × 109 matrix (voxel size: 1.02 × 1.02 × 2.03 mm3) using the built-in ordered subset expectation maximization (OSEM) algorithm with 4 iterations, 21 subsets and a 5 mm Gaussian filter on the Siemens Biograph and with 5 iterations, 24 subsets and a 5 mm Gaussian filter on the Siemens mCT. A low-dose CT scan preceded the PET acquisition and served for attenuation correction. Frame binning (*n* = 35) was standardized to 12 × 5 s, 6 × 10 s, 3 × 20 s, 7 × 60 s, 4 × 300 s and 3 × 600 s.

### Spatial normalization and kinetic modelling

We acquired kinetic parameters using an in-house semi-automated pipeline implemented in PMOD version 4.3 (PMOD Inc), as described previously [10, 26]. In brief, following initial motion correction of each full dynamic dataset, image-derived input functions (IDIF) were obtained through automated extraction of the PET signal from the carotid artery during the 60-minute dynamic PET scan. All images were registered to MNI space using the established [^18^F]PI-2620 PET template [27]. R1 values, indicative of delivery, and K2a efflux rate parameters, representing dissociation from the target, were extracted using SRTM2 as implemented in the Qmodelling package [28]. Distribution volume ratio (DVR) images, reflecting overall tracer binding, and standardized uptake value ratios (SUVR) were calculated using the mean value from a region of interest (ROI) in the inferior cerebellar grey matter as the scaling factor. For ROI analyses, DVRs, R1, and K2a values were extracted in MNI space using delineated regions from the Brainnetome atlas [29]. To determine delivery, tracer efflux or tau-PET abnormality, z-scores were obtained for DVR, R1 and K2a values, using an in-house validated dataset of healthy control subjects (*n* = 17).

### [^18^F]FDG-PET and β-amyloid-PET

Static [^18^F]FDG-PET (30–50 min) and early-phase β-amyloid PET (0–10 min) were normalized to SUVR relative to the mean intensity of a whole-brain volume of interest (VOI). Late-phase β-amyloid PET with [^18^F]Flutemetamol or [^18^F]Florbetaben (90–110 min.) was normalized to SUVR, using the pons and cerebellum as reference regions and was visually assessed by three independent readers (Supplement). Quantitative centiloid values for β-amyloid PET were further derived using the cPET tool provided by Combinostics.

### Visual analysis of stereotactic surface projections

For visual interpretation of early-phase [^18^F]Flutemetamol_0–10 min_/[^18^F]Florbetaben_0–10 min_ (β-amyloid-PET_0–10 min_), [^18^F]PI-2620_0.5–2.5 min_ and [^18^F]FDG-PET images, three-dimensional stereotactic surface projections (3D-SSP) [30] were generated and assessed by three independent readers. A detailed description of the visual assessment of stereotactic surface projections is provided in the Supplement.

### Statistical analysis

Correlations of regional SUVR, R1, K2a, and DVR among imaging ([^18^F]PI-2620, β-amyloid, [^18^F]FDG, cMRI) and cerebrospinal fluid (p-tau-181, t-tau) readouts were evaluated using Pearson’s correlation coefficient (R). Quantitative variables were reported as mean ± standard deviation. An unpaired, two-tailed t-test was used for groupwise comparison of p-tau-181 and t-tau between both tauopathy cohorts. For visual analysis, the inter-reader agreement for [^18^F]PI-2620_0.5–2.5 min_, β-amyloid_0−10 min_ and [^18^F]FDG_30−50 min_ was calculated using Fleiss kappa, while the inter-modality agreement was determined using Cohen’s kappa. A Two-Way Analysis of Variance (ANOVA) was employed including Sidak’s multiple comparisons test for DVR- and K2a z-scores of Brainnetome atlas regions. P values less than 0.0332 were interpreted as statistically significant. P values less than 0.0332, 0.0021, 0.0002, and 0.0001 were shown as *, **, ***, and ****, respectively.

For staging, the CenTauR mask, covering mesial temporal, meta-temporal, posterior cingulate/precuneus, and subfrontal areas [31], was utilized in conjunction with manually delineated PSP target regions [19, 32] in Montreal Neurological Institute (MNI) space, aligned with the tau aggregate patterns identified in histopathological studies as previously described by Kovacs et al. [33]. This approach was used to generate DVR scores, ranging from 0 to 100, by referencing the cases with the lowest and highest uptake in each region across all *n* = 129 participants, respectively. Additionally, the perfusion impairment across the combined CenTauR and Kovacs mask was assessed with the same approach, with a score of 100 representing the case exhibiting the most significant neuronal damage.

An effect size analysis of K2a and DVR scores was performed using the Brainnetome atlas, which includes 246 regions, to identify the top 10% most discriminating regions between the two tauopathy cohorts. The resulting 24 regions were then averaged and used in subsequent correlation analyses with DVR and K2a, as well as with CSF p-tau-181, and to conduct a receiver operating characteristic (ROC) analysis to predict β-amyloid positivity. ROC curves were calculated after logistic regression of K2a and DVR against β-amyloid-PET, CSF and both biomarkers combined. The optimal cutoff for β-amyloid positivity using K2a was estimated using the Youden index. Comparison of the area under the curve (AUC) of ROC curves between K2a and DVR were conducted using a nonparametric approach as previously described by DeLong et al. [34]. A significance level of *p* < 0.05 was applied in all analyses. All statistical analyses were performed using SPSS (version 27.0, IBM, New York, USA) and GraphPad Prism (V9, GraphPad Software).

## Results

### Demographics of the in vivo dynamic [^18^F]PI-2620 tau-PET imaging population

Patients included belong to the AD continuum (total *n* = 47; ⌀75 ± 9 years, 62% female) as 3/4RT or PSP/CBS (*n* = 82; ⌀75 ± 7 years, 43% female) as 4RT and were compared to healthy controls (HC) (*n* = 17; ⌀70 ± 10 years, 53% female). While no significant differences in disease duration were observed between the two groups (*p* = 0.89), patients in the 3/4RT cohort showed significantly lower MoCA scores compared to those in the 4RT group (*p* < 0.0001). PSP Rating Scale scores were not significantly different (*p* = 0.22, Supplementary Fig. 1), which was notably influenced by the inclusion of AD-CBS cases (*n* = 11) in the 3/4RT cohort. Demographics of the study cohort are reported in Table 1.

**Table 1:** Demographics at the group level

Demographics	4RT	3/4RT	Healthy controls
No.	82	47	17
Subgroups	PSP (*n* = 70), CBS (*n* = 12)	MCI (*n* = 11), dementia (*n* = 25), CBS (*n* = 11)	NA
Age, mean (SD), y	75 (7)	75 (9)	70 (10)
Sex, No. (%)			
Female	35 (43)	29 (62)	9 (53)
Male	47 (57)	18 (38)	8 (47)
Dynamic [^18^F]PI-2620 PET, No.	82	47	17
[^18^F]FDG-PET, No.	36	15	NA
β-amyloid-PET, No.	47	31	NA
Centiloid unit, mean (SD)	-8.9 (12.9)	85.8 (23.2)	NA
Volumetric cMRI, No.	43	22	NA
CSF Aβ_42/40_, No.	65	41	NA
CSF p-tau-181 & t-tau, No.	56	39	NA
Plasma NfL, No.	48	11	NA
Disease duration, mean (SD), mo	35.1 (19.8)	34.6 (22.9)	NA
MoCA score, No., mean (SD)	68, 21.7 (4.8)	27, 16.7 (6.2)	NA
PSP RS score, No., mean (SD)	61, 30.6 (12.6)	7, 24.6 (7.4)	NA

Abbreviations: 4RT, 4-repeat tauopathy; 3/4RT, 3/4-repeat tauopathy; CBS, corticobasal syndrome; cMRI, cranial magnetic resonance imaging; CSF, cerebrospinal fluid; FDG, fluorodeoxyglucose; MCI, mild cognitive impairment; mo, months; MoCA, Montreal Cognitive Assessment; NA, not applicable; NfL, neurofilament light chain; PET, positron emission tomography; PSP, progressive supranuclear palsy; PSP-RS, Progressive Supranuclear Palsy Rating Scale; p-tau-181, tau phosphorylated at threonine 181; SD, standard deviation; t-tau, total tau

### K2a images show distinct and specific patterns in tauopathies

**Fig. 2:**
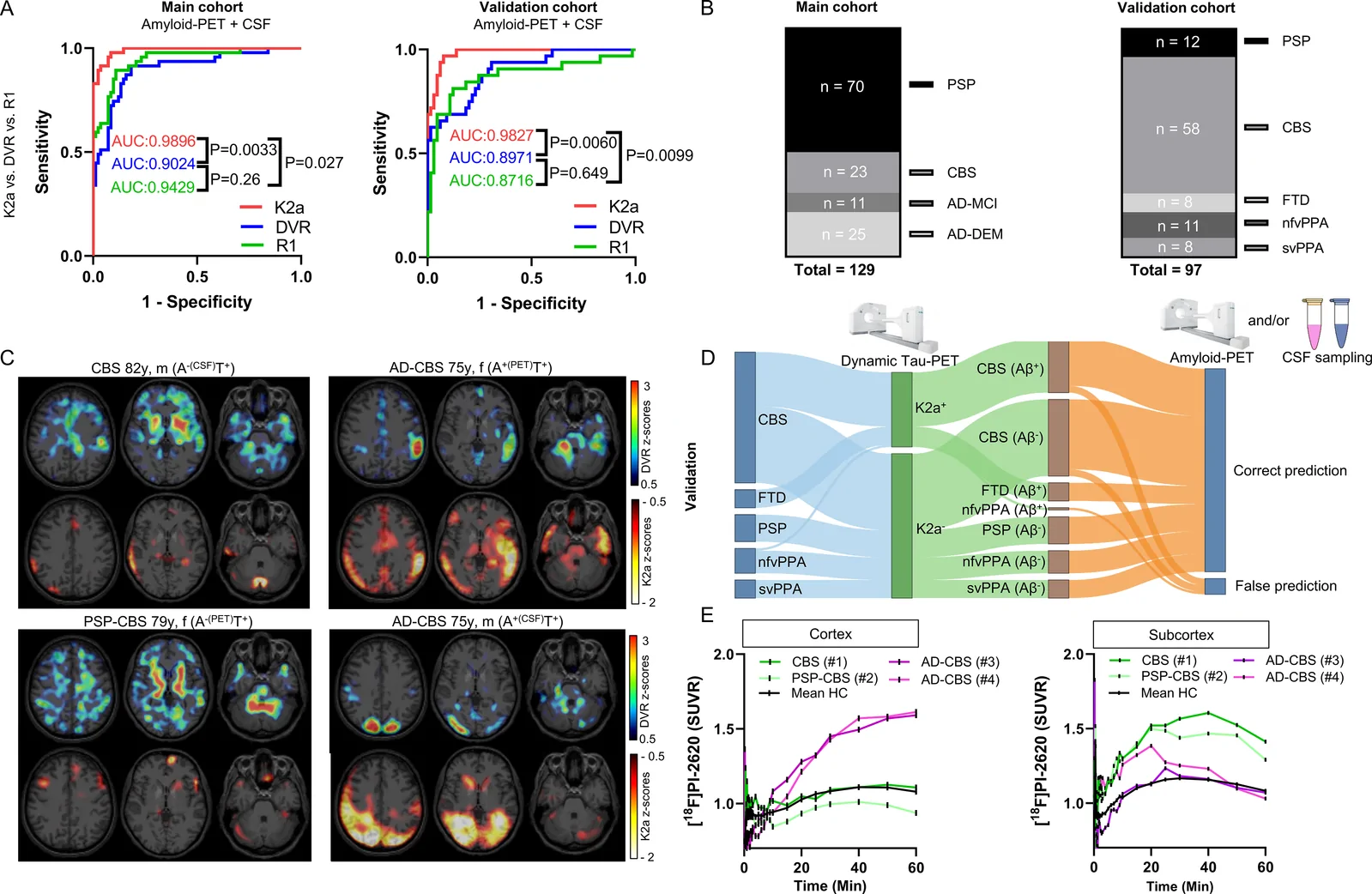
Tau-PET tracer clearance for the prediction of β-amyloid positivity. (**A**) Receiver operating characteristic (ROC) curves comparing the predictive performance of tracer clearance (K2a, red), tau binding (DVR, blue), and perfusion (R1, green) for β-amyloid positivity using the top 10% discriminating regions from the Brainnetome atlas. Performance is shown separately for the main cohort and the validation cohort, defined by combined β-amyloid PET and CSF status. (**B**) Overview of cohort composition. (**C**) Four individuals with clinically probable tauopathies and cortical tau-PET binding were distinguished according to their K2a patterns. Interestingly, off-target binding spots found in the vermal region of the β-amyloid-positive cases did not show significantly altered tracer clearance [35]. (**D**) Sankey diagram illustrating the classification workflow and prediction of β-amyloid status using K2a in the validation cohort. (**E**) Time-SUVR curves for cortex and subcortex in the four representative CBS individuals shown in (**C**) compared with the mean trajectory of healthy controls

First, we compared clearance characteristics of [^18^F]PI-2620 based on K2a, which was successfully derived for all individuals using kinetic modelling [10, 26]. Visual assessment revealed similar K2a maps of 4RT compared to HC with slightly lower tracer clearance in the basal ganglia (Fig. 1A-B). In contrast, 3/4RT (i.e. AD patients) exhibited significantly reduced tracer clearance in cortical regions suggesting strong and persistent tracer binding to AD-type tau, which demonstrated high sensitivity even in individuals with limited late-phase tau-PET signals. In individual β-amyloid-negative patients with PSP-CBS and CBS, no relevant reduction of tracer efflux was observed, even if the basal ganglia exceeded DVR z-scores of 2 (Fig. 2C). In contrast, individual β-amyloid-positive patients with AD-CBS demonstrated elevated DVR in cortical regions. Here, the K2a signal even extended beyond the area of DVR elevation, being widely detectable in distant cortical brain regions (Fig. 2C). Similar results were observed in individuals with AD-MCI, where pronounced K2a signal was found despite being tau-negative on visual read or exhibiting only low tau-load, indicating that K2a may be more sensitive than conventional tau-PET in capturing early pathophysiological changes (Supplementary Fig. 2B-C). A groupwise comparison of CBS patients within the 4RT cohort (*n* = 12) and CBS patients with underlying AD pathology in the 3/4RT cohort (*n* = 11) revealed significantly reduced K2a (*p* < 0.0001) (Supplementary Fig. 3). Of note, we did not observe any significant differences in K2a or DVR between PSP and CBS patients across the investigated subregions of the Brainnetome atlas, supporting the rationale for analyzing these primary 4R tauopathies as a combined group in all subsequent analyses (Supplementary Fig. 4).

### [^18^F]PI-2620 tracer clearance predicts β-amyloid positivity in tauopathies (A)

All K2a z-score values derived from the Brainnetome atlas (*n* = 246), summarized anatomical subregions (*n* = 24), and the 10% most discriminating ROIs (effect size analysis between 4RT and 3/4RT, *n* = 24) are visualized for all individual patients in Fig. 1C. Significant differences of [^18^F]PI-2620 clearance among the two study cohorts were observed in 21 out of 24 subregions. Effect size analysis between both cohorts (4RT versus 3/4RT) revealed highest discriminative power in cortical regions, best represented by the inferior temporal gyrus (Cohen’s d = 2.49) (Fig. 1C-D). 18 out of 24 (75%) of the 10% most discriminative ROIs were identical to the 10% ROIs with the lowest tracer clearance in 3/4RT.

Next, we questioned if quantitative [^18^F]PI-2620 tracer clearance is able to predict the β-amyloid status as assessed by late-phase β-amyloid-PET scans (*n* = 81) and/or CSF Aβ_42/40_ ratio < 5.5% (*n* = 104). In cases with both, Aβ-CSF and Aβ-PET available, we observed a congruency of 85% (22/26) in 3/4RT and 97% (33/34) in 4RT. In cases of discordant Aβ-CSF and Aβ-PET results, the PET measures were used as the decisive read-out [22]. Averaged K2a values of the 24 pre-assessed discriminative ROIs were able to predict the β-amyloid status at a positive predictive value (PPV) of 91.5% and a negative predictive value (NPV) of 95.1% at an AUC of 0.99 (*P* < 0.0001, Fig. 1E). In a sub-analysis of patients with only β-amyloid-PET available, stronger predictive performance was observed, with a PPV of 93.6%, an NPV of 96.0%, and an AUC of 0.99 (*P* < 0.0001). This exceeded the performance in the CSF-only sub-analysis (PPV: 88.6%, NPV: 88.4%, AUC: 0.94; *P* < 0.0001; Fig. 1E).

To further challenge its added value, we compared the predictive performance of K2a with tau binding (DVR) and perfusion (R1). K2a showed significantly stronger discrimination of β-amyloid positivity by combined PET/CSF assessment (vs. DVR: *P* = 0.0033; vs. R1: *P* = 0.027) (Fig. 2A). Importantly, we validated these findings in an independent and clinically heterogeneous validation cohort (*n* = 97), comprising challenging neurodegenerative syndromes (Fig. 2B, Supplementary Table 1). Within this cohort, K2a-based prediction of β-amyloid status consistently outperformed DVR and R1, confirming its superior predictive performance (Fig. 2A). Furthermore, K2a-based classification yielded strong positive and negative predictive values (PPV: 87.9%, NPV: 95.3%) and an AUC of 0.98 (*P* < 0.0001), underscoring its robustness for predicting β-amyloid positivity across diverse clinical phenotypes (Fig. 2A, D).

To support the kinetic interpretation underlying these findings, we additionally analyzed time-SUVR curves in four representative individuals with CBS, including two β-amyloid-positive AD-CBS and two β-amyloid-negative 4R cases. In cortical regions, 3/4R cases exhibited continuously increasing time-SUVR curves, while 4R cases showed plateauing or declining trajectories, consistent with faster tracer washout. Subcortically, all curves followed a bell-shaped pattern. These visual kinetic differences further support the mechanistic rationale for K2a (Fig. 2C, E).

Furthermore, reduced K2a showed a significant association with Centiloid units in 3/4RT when considering the top 10% of discriminating regions (*R*=-0.45, *P* = 0.014) or the CenTauR Meta Temporal ROI (*R*=-0.42, *P* = 0.021) (Supplementary Fig. 5).

### [^18^F]PI-2620 binding indicates distinct topology among different tauopathies (T)

**Fig. 3:**
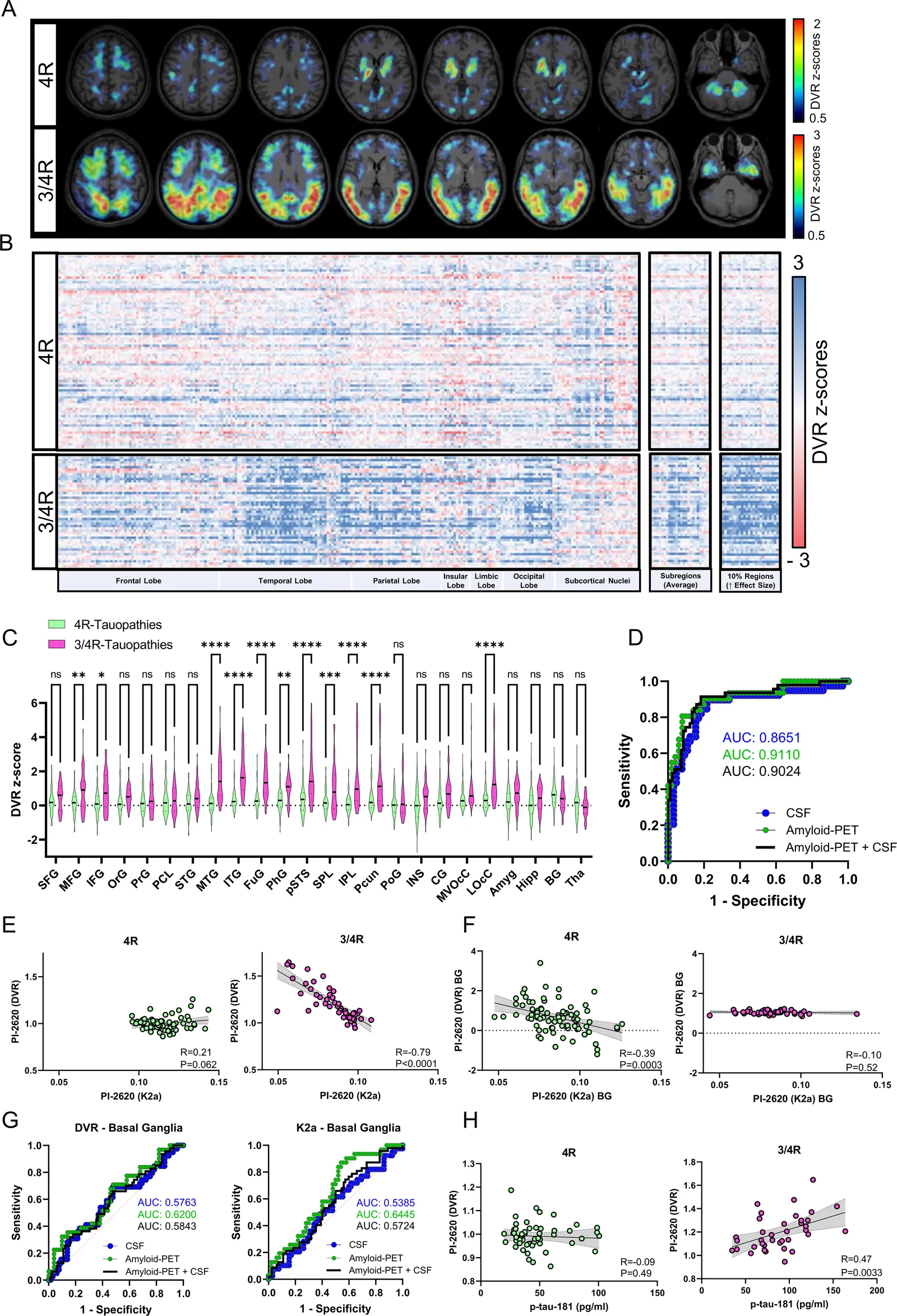
[^18^F]PI-2620 binding for the detection of tau accumulation patterns in 4RT and 3/4RT. (**A**) Average [^18^F]PI-2620_DVR_ z-score maps of 4RT and 3/4RT, illustrated by axial slice overlays on a standard MRI template in MNI space. (**B**) Heatmaps on the left show DVR z-scores of all Brainnetome atlas regions for all individuals. Heatmaps on the right show the top 10% of discriminating regions determined by effect size analysis between both cohorts. Intermediate heatmaps illustrate the composite Brainnetome atlas subregions. (**C**) Two-way ANOVA analysis of Brainnetome atlas subregions, *p < 0.0332, **p < 0.0021, ***p < 0.0002. ****p < 0.0001. (**D**) ROC curves represent the results of a logistic regression analysis using DVR of the top 10% discriminating regions against CSF (blue dots), β-amyloid-PET (green dots), and both combined (black line) to predict β-amyloid positivity in all patients. (**E-F**) Correlation plots visualize association between [^18^F]PI-2620 K2a and DVR in the 10% most discriminating K2a-based regions and the basal ganglia solely. (**G**) ROC analysis of [^18^F]PI-2620 K2a and DVR derived from the basal ganglia, validated against β-amyloid gold standard assessments. (**H**) Scatterplots depict association between [^18^F]PI-2620_DVR_ and p-tau-181 from CSF in both study cohorts

As expected from our previous research [10], significant differences in [^18^F]PI-2620 binding among both study cohorts, utilizing DVR, were observed in 11/24 Brainnetome subregions, all located within the cortex. Interestingly, the regional effect size analysis again revealed the highest difference among all ROIs in the inferior temporal gyrus (Cohen’s d = 1.85, compared to d = 2.49 for K2a) (Fig. 3A-C). Again, a ROC analysis was performed, now using the averaged DVR of the top 10% of ROIs with the highest discriminatory power to test whether [^18^F]PI-2620_DVR_ successfully predicts β-amyloid positivity. AUC of DVR β-amyloid status prediction was 0.91 (*P* < 0.0001) for validation against β-amyloid-PET, 0.87 (*P* < 0.0001) for CSF and 0.90 (*P* < 0.0001) for both modalities combined (Fig. 3D).

To better understand the proposed biomarker of tracer clearance, we explored the association between [^18^F]PI-2620 tau binding and K2a in the predefined K2a-based discriminatory ROIs. While DVR and K2a only showed a trend towards correlation in patients with 4RT (*R* = 0.21; *P* = 0.062), a strong negative association between tau binding and tracer clearance was observed in patients with 3/4RT (*R*=-0.79; *P* < 0.0001) (Fig. 3E). In the basal ganglia, K2a was negatively associated with DVR in patients with 4RT (*R*=-0.39; *P* = 0.0003), but not in patients with 3/4RT (*R*=-0.10; *P* = 0.52) (Fig. 3F). ROC analysis of K2a in the basal ganglia revealed significant prediction of β-amyloid PET positivity as a gold standard (AUC = 0.64; *P* = 0.030), but not when β-amyloid in CSF (AUC = 0.54; *P* = 0.51) or both assessments (AUC = 0.57; *P* = 0.17) were considered for determination of the β-amyloid status. Similarly, [^18^F]PI-2620 DVR in the basal ganglia did not show significant prediction of the β-amyloid status in PET (AUC = 0.62; *P* = 0.07), CSF (AUC = 0.58; *P* = 0.19), or when assessed by both biomarkers (AUC = 0.58; *P* = 0.11) (Fig. 3G). There was no significant correlation between DVR and p-tau-181 in the discriminatory regions of 4RT (*R*=-0.09; *P* = 0.49), but a significant correlation in the 3/4RT group (*R* = 0.47; *P* = 0.0033) (Fig. 3H), along with the overall elevated p-tau-181 levels in 3/4RT compared to the 4RT group (*P* < 0.0001) (Supplementary Fig. 6).

### Delivery of [^18^F]PI-2620 for detection and staging of neuronal injury in tauopathies (N)

Early-phase [^18^F]PI-2620 has shown promising results in identifying neuronal injury in patients with tauopathies, demonstrating correlations with established indices of neurodegeneration in small mixed samples [15] and providing intriguing insights in 4RT [16]. Additionally, early-phase tau-PET provides similar regional information compared to early-phase β-amyloid-PET [36], further supporting its role in diagnostic algorithms [10]. However, despite these encouraging findings, the application of [^18^F]PI-2620 has not yet been systematically validated in larger cohorts and in conjunction with β-amyloid and tau assessment. Thus, we aimed to comprehensively evaluate the utility of [^18^F]PI-2620 in this A/T/N evaluation of patients with tauopathies. As part of this framework, we also evaluated which PET-derived parameter best reflects neurodegeneration (N). We focused on R1 as a surrogate for neuronal injury and found that R1 outperformed both DVR and K2a in predicting N status, as defined by consensus visual assessment of early-phase PET signals (Supplementary Fig. 7). These findings support R1 as the most reliable tau-PET–derived marker of neurodegeneration. Further insights into its regional behaviour are provided in the Supplementary Results and Supplementary Figs. 8–11.

**Fig. 4:**
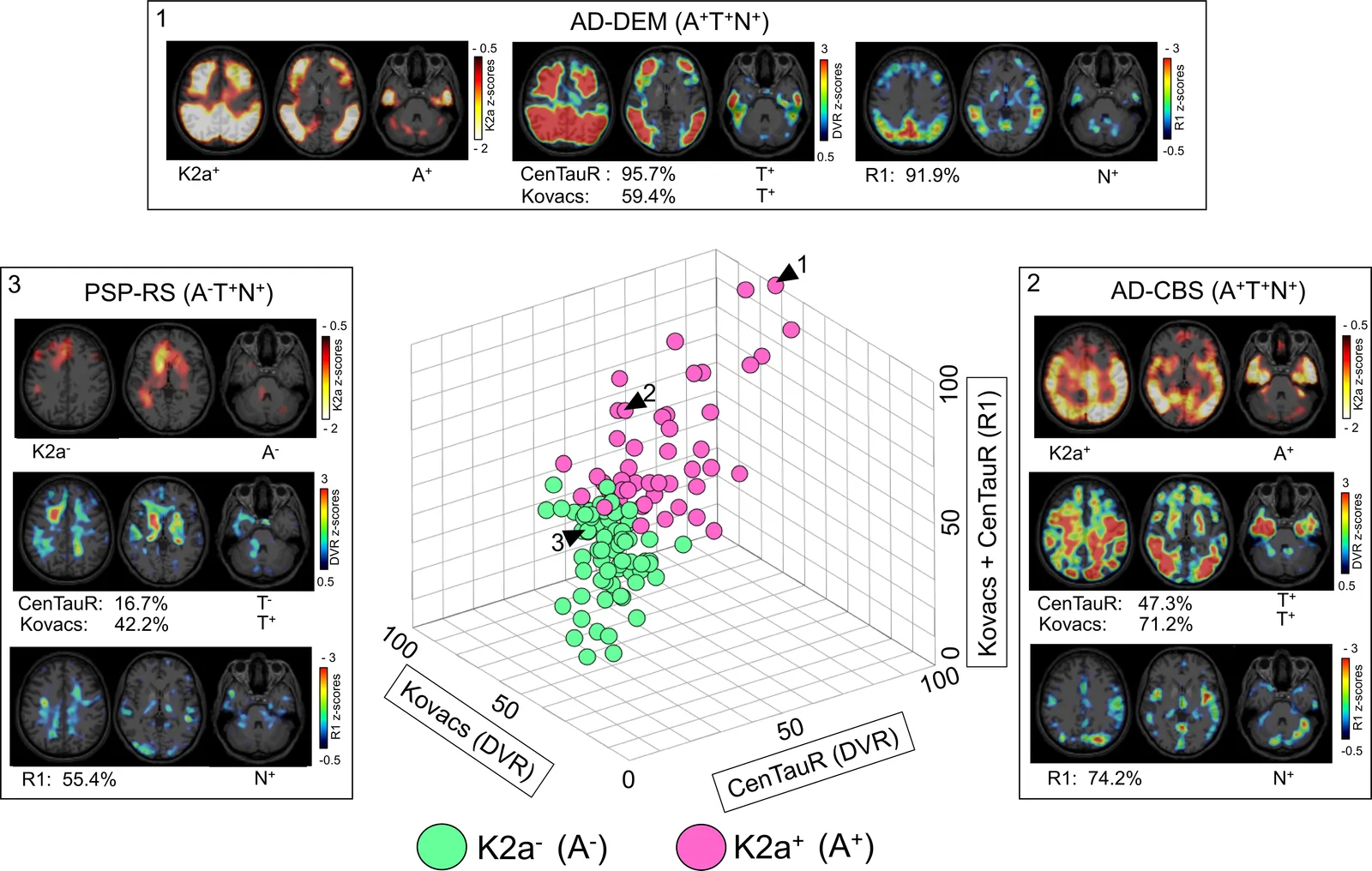
3-dimensional quantitative staging determines personalized trajectories of A/T/N-based disease severity in patients with tauopathies. Axial slices of representative patients with tauopathies with varying tau burden in the CenTauR and Kovacs masks are shown alongside K2a and R1 z-score images. The percentage values are calculated based on scaling relative to the individual with the highest and lowest values. 3D scatter plot visualizes all *n* = 129 participants, with K2a predicted β-amyloid-positivity (purple dots) and β-amyloid-negativity (green dots)

To assess the potential of tracer delivery in staging neuronal damage, we examined tracer perfusion within a combined global mask including AD (CenTauR) [37] and PSP (Kovacs) [19, 33] target regions. This assessment was performed alongside with staging of the individual patient’s tau burden, as obtained by evaluation of tau-PET DVR for each of the two distinct sets of composite regions. After categorizing participants as β-amyloid-positive or negative according to the K2a cutoff determined by Youden’s index, a 3D scatter plot was generated, which effectively visually distinguished AD cases from primary tauopathies. The analysis of exemplary cases further illustrated these findings, with tau-PET binding patterns in cortical regions contributing to a higher CenTauR score, while enhanced uptake in the basal ganglia was associated with a higher Kovacs score (Fig. 4). The results of the entire cohort suggested that 3-dimensional quantitative staging can determine personalized trajectories of A/T/N-based disease severity (Fig. 4).

A subset of both cohorts received additional [^18^F]FDG-PET_30−50 min_ (4RT, *n* = 36; 3/4RT, *n* = 15), β-amyloid-PET0-10 min (4RT, *n* = 37; 3/4RT, *n* = 26) and CSF analysis with total-tau (t-tau) examination (4RT, *n* = 56; 3/4RT, *n* = 39), which served for gold standard validation of early-phase [^18^F]PI-2620 assessment as a surrogate of neurodegeneration. The intermodal correlation analysis revealed significant relationships across all major regions studied. The strongest correlations were observed between early-phase [^18^F]PI-2620 and [^18^F]FDG-PET (*R* = 0.53, *P* < 0.0001), as well as between early-phase [^18^F]PI-2620 and β-amyloid-PET_0−10 min_ (*R* = 0.56, *P* < 0.0001) in the parietal cortex (Supplementary Results, Supplementary Fig. 8). To further assess clinical applicability, we additionally performed a visual read of early-phase [^18^F]PI-2620 images and compared it with established modalities. Visual agreement across readers and modalities was high, supporting early-phase tau-PET as a robust surrogate marker of neurodegeneration. Detailed results of the inter-reader and inter-modality agreement analyses are provided in the Supplementary Material (Supplementary Results, Supplementary Fig. 10).

Evaluation of t-tau indicated an elevation in the 3/4R cohort compared to the 4RT (*p* < 0.0001), along with a negative agreement with whole brain [^18^F]PI-2620_R1_ in 4RT (*R*=-0.29; *P* = 0.031) and 3/4RT (*R*=-0.35; *P* = 0.031) (Supplementary Fig. 6, 11). A comparison of plasma NfL in a subset of participants revealed elevated levels in 3/4RT compared to 4RT (*P* = 0.014) (Supplementary Fig. 6).

In conclusion, quantitative staging of ATN with a single dynamic [^18^F]PI-2620 acquisition could enable detailed characterization of the individual patient’s biomarker trajectory.

## Discussion

Molecular imaging is becoming increasingly prominent in definition schemes of neurodegenerative diseases, particularly AD [20] but also for movement disorders [38, 39]. The evolving diagnostic criteria for AD emphasize the use of a single Core 1 biomarker for biological diagnosis across the disease continuum [20]. This biological-centric approach aligns with the broader A/T/N classification but raises questions about its clinical implications. Recent critiques argue that focusing solely on β-amyloid (A) positivity may overlook the complexity of tau (T) and neurodegeneration (N) biomarkers, potentially leading to premature diagnoses in asymptomatic individuals [40, 41]. These concerns underscore the importance of integrating a comprehensive A/T/N assessment, rather than relying on a single biomarker, to capture the dynamic interplay of β-amyloid, tau, and neurodegenerative processes. Our findings support this holistic view, demonstrating that multi-faceted tau-PET metrics provide a robust framework for disease staging and progression monitoring. Therefore, it is essential to establish discriminative biomarkers that characterize the disease trajectory of the individual patient by extracting the maximum information from each PET examination to minimize cost and burden to individual patients and the healthcare system.

To this end, we exploited the value of dynamic [^18^F]PI-2620 tau-PET imaging to decipher the entire A/T/N classification scheme in patients with distinct tauopathies with a single scan in a cross-sectional study design. We demonstrated that assessment of the tracer efflux via [^18^F]PI-2620_K2a_ is a highly effective kinetic PET-biomarker for distinguishing between 3/4RT and 4RT patients. Furthermore, we highlight the significance of [^18^F]PI-2620_DVR_ to quantify regional tau burden, and in conjunction with [^18^F]PI-2620_R1_, a biomarker of neuronal injury, for detailed disease staging.

As the main finding of our study, we observed similar regional [^18^F]PI-2620 efflux for 4RT patients and healthy controls, with only minor alterations observed in the basal ganglia region, whereas, 3/4RT patients exhibited significantly reduced tracer clearance in cortical regions. This differential efflux pattern can be explained by the distinct tau isoform-specific characteristics of [^18^F]PI-2620 binding and clearance. According to previous findings, the K2a pattern varies with tau isoform composition. In 4RT, efflux patterns resemble healthy controls due to lower binding affinity or reduced entrapment in 4R tau aggregates. In contrast, 3/4RT show higher tracer retention and reduced cortical clearance, reflecting complex binding dynamics from mixed tau isoforms [12]. Therefore, lower K2a values align with the pathological profile characteristic of AD, highlighting that the presence of both β-amyloid and tau biomarkers, specifically 3/4R tau, is essential for a reliable AD diagnosis [20]. When we challenged [^18^F]PI-2620_K2a_ in a ROC analysis against DVR, the highest discriminatory power for prediction of β-amyloid-positivity was obtained for the presented clearance biomarker. When compared against the gold standards of binary β-amyloid-PET scans and CSF Aβ_42_/Aβ_40_ ratios [42, 43], [^18^F]PI-2620_K2a_ showed high positive and negative predictive values, indicating its strong potential for accurate classification of the β-amyloid status.

Importantly, these findings were confirmed in an independent validation cohort comprising a clinically heterogeneous spectrum of neurodegenerative syndromes, including phenotypes of CBS and PPA that are known to harbor distinct underlying neuropathologic changes. In contrast to the main cohort, the validation cohort was additionally mixed with respect to tau-PET positivity, thereby providing a more stringent and clinically realistic test of K2a-based β-amyloid prediction. Despite increased clinical variability, K2a retained its superior performance for predicting β-amyloid positivity compared to DVR and R1, highlighting its robustness and generalizability across different disease entities. Notably, regions and thresholds defined in the main cohort were applied without re-optimization of model parameters, yet K2a-based classification still achieved high AUC as well as strong positive and negative predictive values in this cohort, underscoring its potential for reliable application in real-world clinical settings.

Our findings align with several studies in which [^18^F]PI-2620 has demonstrated higher PET signals in patients with 3/4RT compared to patients with 4RT [10, 19, 21, 44, 45], owing to its more persistent and energetically favorable tracer kinetic profile in the presence of 3/4R tau filaments. As the underlying mechanism of action there is evidence from (i) in vitro binding assays, (ii) molecular docking studies [11], and (iii) autoradiography that [^18^F]PI-2620 has stronger binding affinity to 3/4R-tau compared to 4R-tau isoforms [8, 46]. This is further supported by recent findings of molecular dynamics and Brownian dynamics simulation, which revealed that 3/4R tau filaments, particularly within the C-shaped groove of AD tau, offer multiple high-affinity surface binding sites and elevated association rates. In contrast, 4R tau filaments exhibit fewer surface-accessible and energetically stable binding pockets, leading to weaker and more transient interactions [11]. As a result, even in the presence of significant tau pathology, 4RT show lower tracer retention, highlighting that the observed PET signal is not merely a function of tau quantity, but also of isoform-specific binding kinetics. In this regard, structural biology studies using cryo-electron microscopy have further emphasized the conformational diversity of tau filaments across diseases, providing a biological rationale for the observed tracer behavior and supporting the isoform-sensitive properties of [^18^F]PI-2620 [47–49].

In addition to different kinetic profiles, we also exploited the different topologies of tau accumulation. In line with previously published data, 4RT showed mainly subcortical tau accumulation, particularly in the globus pallidus [19, 21, 32, 33], whereas 3/4RT patients had predominant cortical PET signals. Previous efforts to standardize quantitative tau measurements across different tracers have supported the development of a universal scale. Notable examples include CenTauR, introduced for the Alzheimer’s disease continuum [37], and histopathologically defined regions for PSP as outlined by Kovacs et al. [33]. As a major novelty, we provide the first proof of concept for staging distinct tauopathies within the A/T/N framework. To this end, we combined established disease-specific composite regions, CenTauR for AD and Kovacs-defined regions for PSP, with PET-derived surrogate markers: K2a as a proxy for β-amyloid status, DVR as a measure of tau burden, and R1 as an indicator of neuronal injury. These anatomical templates, originally developed from histopathological or tau-PET based studies, were adapted to dynamic imaging and applied to each subject. By plotting individual participants across two axes representing regional tau accumulation and one axis reflecting perfusion deficits, we constructed a 3-dimensional staging index that allowed for a clear visual and quantitative separation of 3/4RT and 4RT cases. Subjects were further stratified by β-amyloid status using a K2a-based cutoff, resulting in individualized A/T/N profiles. This ability to differentiate tauopathies in such detail lays the groundwork for a deeper understanding of the underlying mechanisms, particularly in the context of regional tau aggregation patterns. While these results demonstrate the feasibility of individualized disease staging using dynamic tau-PET, we acknowledge that further validation in larger and independent cohorts will be necessary to establish generalizable cutoffs and confirm robustness across different scanners and clinical settings.

However, when examining specific regions like the basal ganglia, we observed only a trend towards a higher [^18^F]PI-2620_DVR_ in the basal ganglia of 4RT compared to AD, which did not remain significant after multiple comparison testing. This finding may be attributed to the distinct binding characteristics of [^18^F]PI-2620 to different tau isoforms, where even a low deposition of 3/4R tau in the basal ganglia of AD (e.g., AD-CBS) could generate a higher PET signal than the substantial 4R tau deposition observed in PSP cases [19, 50]. Importantly, the inclusion of CBS cases in both 3/4R and 4R groups served as a deliberate design feature to challenge the ability of dynamic tau-PET to resolve underlying tau isoform composition within a shared clinical phenotype. CBS is known to represent a pathologically heterogeneous syndrome, encompassing both AD-type 3/4R and 4R tauopathies [4]. This made it an ideal model to evaluate whether kinetic parameters such as K2a can reliably differentiate tau isoforms even when clinical presentation is overlapping. Our findings confirm that, despite similar clinical presentations and comparable tau positivity on visual reads, K2a quantitatively and visually distinguished CBS cases according to their underlying molecular subtype. This reinforces the utility of K2a as an isoform-sensitive marker capable of resolving tau heterogeneity within clinically overlapping phenotypes. This also contributed to atypical clinical scores within our cohort, reflecting the heterogeneity particularly present in the AD group. For instance, PSP-RS scores within the 3/4R tau cohort were entirely driven by individuals with AD-CBS, a clinical phenotype known to exhibit higher PSP-RS values despite underlying Alzheimer’s pathology. Consistent with our previous findings, the inclusion of CBS cases in the 4RT cohort led to a slight reduction in the overall average PSP-RS score. CBS patients tend to present with lower PSP-RS scores compared to typical PSP cases, who constituted the majority of the 4RT group in this study and showed the expected higher scores [10, 21].

A second ROC analysis further validated the discriminatory power of [^18^F]PI-2620 DVR with high potential to distinguish β-amyloid positivity [10]. In addition, we also tested the predictive performance of the R1 parameter, which interestingly showed a similar performance to DVR. Notably, the comparison between the ROC analyses of K2a, DVR, and R1 highlighted the superior performance of K2a in discriminating β-amyloid-positivity. This finding suggests that K2a may provide a more nuanced and sensitive measure of β-amyloid-related changes. Although the visual appearance of K2a maps differs markedly from that of traditional β-amyloid PET images, this is mechanistically expected, as K2a reflects tracer clearance dynamics rather than direct β-amyloid binding. Importantly, this surrogate relationship is functionally rooted in the β-amyloid-dependent formation of AD-type tau and was confirmed through correlations with Centiloid values and predictive classification metrics. To further illustrate the kinetic mechanisms underlying this improved performance, we analyzed time-SUVR curves from representative individuals with cortical tau-PET uptake, including both AD-related CBS and β-amyloid-negative 4R CBS cases. These curves revealed a clear distinction between phenotypes: in cortical regions, AD-CBS cases showed a continuous SUVR increase throughout the scan duration, indicating persistent tracer binding, while 4R tauopathy cases exhibited plateauing or even declining SUVR trajectories, consistent with faster washout.

A useful analogy is provided by phosphorylated tau biomarkers in fluid. Although p-tau181 and p-tau217 are tau-derived markers, they show high specificity for AD-type pathophysiology and are strongly associated with Aβ positivity, whereas primary 4R tauopathies such as PSP and CBD often show reduced p-tau levels compared with AD [51–54]. Consistently, we observed elevated CSF p-tau181 in AD compared with 4RT. Thus, tau-related biomarkers can behave as AD-specific markers when they reflect the Aβ-associated molecular context of AD-type tau pathology rather than tau aggregation in general. Similarly, K2a is not interpreted as a direct measure of β-amyloid binding, but as a kinetic tau-PET parameter whose predictive value reflects AD-specific tracer-tau interactions and the more persistent binding characteristics of mixed 3/4R tau filaments.

To further evaluate the sensitivity of K2a in atypical and early disease presentations, we analyzed β-amyloid–positive individuals who displayed either visually negative or only minimally elevated tau-PET signals, such as those in early-stage AD-MCI. Interestingly, several of these A^+^/T^–^ cases already exhibited pronounced K2a alterations, despite absent or subtle DVR changes. This supports the notion that K2a may detect early pathophysiological changes related to AD-type tau aggregation that are not yet visually appreciable on conventional tau-PET. However, we also identified individuals with β-amyloid positivity but entirely negative K2a and DVR signals, suggesting that a minimal threshold of tau pathology may be required for K2a sensitivity.

Additionally, we provide evidence that [^18^F]PI-2620_DVR_ and [^18^F]PI-2620_K2a_ are strongly negatively associated in cortical regions of 3/4RT, but not in 4RT patients. The opposite was observed in the basal ganglia, highlighting the dependence of [^18^F]PI-2620 binding affinity on actual tau abundance in the target tissue. Consequently, we questioned if the sole consideration of the basal ganglia could successfully predict β-amyloid positivity in the evaluated patients with tauopathies. The ROC analysis of K2a in the basal ganglia revealed that significant discrimination was achieved only with PET validation, however, this was not replicated with CSF or combined biomarker validation. Conversely, DVR did not demonstrate significant discriminatory power in the basal ganglia, suggesting that this region may be less effective for β-amyloid status prediction using [^18^F]PI-2620 binding.

As the third pillar of the proposed staging scheme, our study provides valuable insights into the utility of [^18^F]PI-2620 tracer delivery as a predictive marker for neuronal damage in patients with tauopathies. We exploited concomitant scans of [^18^F]FDG-PET and early-phase β-amyloid-PET, as well as CSF analysis with total-tau evaluation for gold-standard validation of early-phase tau-PET as a regional surrogate biomarker of neurodegeneration [15, 55, 56].

In line with literature, our findings showed a significant association between [^18^F]PI-2620_R1_, [^18^F]FDG-PET and early-phase β-amyloid-PET [25]. Likewise, analysis of CSF total-tau suggested an inverse relationship between whole brain perfusion and neuronal injury [57]. We also observed a significant association between [^18^F]PI-2620_R1_ and volumetric cMRI, particularly in the 3/4RT [58] and less pronounced in the 4RT group [59, 60]. Interestingly, subcortical analysis demonstrated divergent relationships between [^18^F]PI-2620_R1_ and atrophy in specific brain structures, consistent with previous studies reporting reduced volumes of the nucleus caudate [61] and thalamus [62, 63]. In the globus pallidus, we observed a negative relationship between R1 and atrophy, consistent with previous reports of enhanced perfusion in 4RT [16] and AD [64, 65]. Although the mechanisms underlying these perfusion alterations are not yet fully understood, it is hypothesized that regions affected by atrophy may exhibit compensatory pathological increases in neuronal activity and neuroinflammation, resulting in elevated perfusion depending on the disease stage [66].

Finally, visual assessments of early-phase tau-PET maps revealed excellent inter-reader and inter-modality agreements. These results emphasize the reliability and consistency of visual assessments across various PET modalities and underscore the value of early-phase tau-PET imaging as a surrogate of cerebral perfusion in patients with tauopathies, with clinical implications for diagnosis and staging.

This study has several limitations, including, despite a few cases, the absence of autopsy confirmation for the clinically diagnosed cases, which could have led to diagnostic inaccuracies. The primary aim of this study was to demonstrate the feasibility of classifying individual patients within the A/T/N classification scheme using dynamic tau-PET imaging. We calculated DVR and K2a thresholds through ROC analysis, establishing cutoffs that allowed for assignment into either the β-amyloid-positive or β-amyloid-negative group. Through further quantitative regional assessment of tau load and perfusion, we were able to differentiate primary and secondary tauopathies (e.g., AD-CBS vs. 4R-CBS), which allowed us to successfully categorize and stage individuals. As a limitation, we acknowledge that our staging scheme is a preliminary approach, warranting further advancement by additional healthy controls and larger populations. Normalizing and harmonizing results across multicenter settings, similar to the Centiloid scale for β-amyloid imaging [67], will be essential. Defining cut-offs based on larger cohorts will improve the ability to discriminate between different neurodegenerative disorders. While we focused on ROI-based analyses to capture biologically and clinically meaningful patterns, future studies may benefit from voxelwise cross-modal comparisons, using Parametric Mapping, to further explore spatial relationships between tau pathology, neurodegeneration, and corresponding surrogate kinetic parameters such as R1 and DVR.

Our results demonstrate that the accurate assessment of altered K2a is effective only in the presence of established tau pathology. Consequently, K2a is likely to lose sensitivity in the very early stages of AD. Further longitudinal studies are needed to address this gap and may capture changes in neurofibrillary profiles and the recently proposed shifts in tau isoforms throughout the trajectory of AD in vivo [68–70]. Nevertheless, even in individuals with a visually negative [^18^F]PI-2620 scan, relevant information can still be extracted from the early-phase signal via the R1 parameter. Since R1 reflects relative tracer delivery and is not influenced by specific tau binding, it remains a valid marker of perfusion and neurodegeneration independent of T status. This suggests that, even in the absence of detectable tau pathology, dynamic tau-PET may still contribute valuable information regarding N status.

## Conclusion

In summary, dichotomous assessment at the individual patient level revealed a strong agreement between semiquantitative and visual methods in reliably distinguishing patients with clinically diagnosed 3/4RT from those with 4RT. Importantly, this study underscores the potential of dynamic [^18^F]PI-2620 tau-PET imaging to classify and stage patients within the A/T/N framework, providing valuable insights for future clinical trials. This approach suggests that a single dynamic [^18^F]PI-2620 tau-PET may reduce the need for additional β-amyloid- and [^18^F]FDG-PET examinations in selected diagnostic settings, thereby reducing radiation exposure for patients and lowering healthcare costs.

## Supplementary Information

Below is the link to the electronic supplementary material.

Supplementary Material 1

## Data Availability

The datasets generated during and/or analyzed during the current study are available from the corresponding author on reasonable request.

## Declarations

### Author information

These authors jointly supervised this work: Günter Höglinger, Nicolai Franzmeier, Matthias Brendel.

## Abbreviations

3/4RT: 3/4-repeat tauopathy
3D-SSP: Three-dimensional stereotactic surface projection
4RT: 4-repeat tauopathy
A/T/N: Amyloid/tau/neurodegeneration
Aβ: β-amyloid
AD: Alzheimer’s disease
AUC: Area under the curve
CBS: Corticobasal syndrome
CBD: Corticobasal degeneration
cMRI: Cranial magnetic resonance imaging
CSF: Cerebrospinal fluid
CT: Computed tomography
DVR: Distribution volume ratio
ELISA: Enzyme-linked immunosorbent assay
[^18^F]FDG: [^18^F]fluorodeoxyglucose
FTD: Frontotemporal dementia
HC: Healthy controls
HPLC: High-performance liquid chromatography
IDIF: Image-derived input function
K2a: Apparent tissue efflux rate constant
MCI: Mild cognitive impairment
MNI: Montreal Neurological Institute
MoCA: Montreal Cognitive Assessment
MRI: Magnetic resonance imaging
NfL: Neurofilament light chain
NPV: Negative predictive value
OSEM: Ordered-subset expectation maximization
PET: Positron emission tomography
PPV: Positive predictive value
PSP: Progressive supranuclear palsy
PSP-RS: Progressive Supranuclear Palsy Rating Scale
p-tau-181: Tau phosphorylated at threonine 181
R1: Relative tracer delivery
ROC: Receiver operating characteristic
ROI: Region of interest
SD: Standard deviation
SRTM2: Simplified reference tissue model 2
SUVR: Standardized uptake value ratio
t-tau: Total tau
VOI: Volume of interest
