# Supplementary material for: Dynamic [18F]PI-2620 recording facilitates prediction of β-amyloid positivity and simultaneous staging of tau and neurodegeneration
Source: Mol Neurodegener. 2026 Sep 24;21:59. doi: 10.1186/s13024-026-00997-3 (PMC13615581; doi:10.1186/s13024-026-00997-3)
Supplement: Supplementary file 1 [file 13024_2026_997_MOESM1_ESM.docx]

**Supplementary Information**

**Supplementary Methods**

| Demographics | PSP | CBS | FTD |
| --- | --- | --- | --- |
| No. | 12 | 58 | 27 |
| Age, mean (SD), y | 68.8 (9.4) | 69.1 (8.6) | 68.1 (8.6) |
| Female | 6 | 32 | 16 |
| Male | 6 | 26 | 11 |
| Dynamic [^18^F]PI-2620 PET, No. | 12 | 58 | 27 |
| Tau-PET, % pos. | 83.3 | 79.3 | 55.6 |
| β-amyloid-PET, No. | 7 | 24 | 13 |
| CSF Aβ_42/40_, No. | 11 | 48 | 23 |
| β-amyloid, % pos. | 0 | 41.4 | 29.6 |

**Supplementary Table 1. Demographics of the validation cohort at the group level.**

*Visual analysis of late-phase β-amyloid PET images*

Late-phase β-amyloid PET scans (90-110 min p.i.) with [^18^F]flutemetamol and [^18^F]florbetaben were visually assessed by three experienced nuclear physicians (J.G., P.B., and M.B.) following manufacturer guidelines (Life Molecular Imaging for NeuraCeq, GE HealthCare for Vizamyl) as described previously [1]. In brief, images were classified as positive (binding in one or more cortical brain regions) or negative (predominantly white matter uptake). All images were systematically evaluated from the pons/cerebellum upwards through the frontal lobes, anterior cingulate, posterior cingulate, precuneus, temporoparietal regions (including the insula and lateral temporal lobes), and the striatum. Visual interpretation was based on comparing tracer uptake in cortical grey matter with adjacent white matter. Readers manually adjusted the threshold window using the pontine/cerebellar white/grey matter contrast as a reference. Semiquantitative Aβ-PET data, expressed as SUVR and z-scores in the target regions and their composite, were available as supporting information. The pons was used as the reference tissue for [^18^F]flutemetamol, while the cerebellar cortex served as the reference for [^18^F]florbetaben. PET images were analyzed using a rainbow color scale for [¹⁸F]flutemetamol and a grayscale for [^18^F]florbetaben. In cases of discrepancies, the final classification was determined by consensus. All readers had access to relevant clinical information and structural imaging (MRI ~70%, CT ~30%) to aid interpretation.

*Visual analysis of stereotactic surface projections*

For visual interpretation of early-phase [^18^F]Flutemetamol_0–10min_/[^18^F]Florbetaben_0–10min_ (β-amyloid-PET_0–10min_), [^18^F]PI-2620_0.5–2.5min_ and [^18^F]FDG-PET images, three-dimensional stereotactic surface projections (3D-SSP) [2] were generated using the software Neurostat (Department of Radiology, University of Washington, Seattle, WA, USA). Voxel-wise Z-scores were calculated in Neurostat by comparing the individual tracer uptake of β-amyloid-PET_0–10min_, [^18^F]PI-2620_0.5–2.5min_ and [^18^F]FDG to a historical [^18^F]FDG-PET database (cognitively healthy individuals, age-matched, n=18). Three experienced nuclear medicine physicians (J.G., P.B., and M.B.) visually assessed the 3D-SSP images using the Z-score maps (GBM scaling) and rated the severity of perfusion and metabolic reduction in cortical regions used in the clinical routine (frontal, parietal, temporal, occipital cortex areas) from 0 to 3 (0 = no reduction, 1 = mild, 2 = intermediate, 3 = severe). Based on the individual readers assessment, whole-brain 3D-SSP images were rated binary (0/1) and according to the severity of present neurodegeneration (0 = no/1 = mild/2 = intermediate/3 = severe neurodegeneration). Readers had access to the pre-PET clinical diagnosis and to the scan modality. Prior to the visual read, all three readers received detailed instructions including 10 training sets. Reading was performed in balanced order to avoid systematic bias. Reader 1 started with [^18^F]FDG-PET, continued with β-amyloid-PET and [^18^F]PI-2620_0.5–2.5min_; Reader 2 started with [^18^F]PI-2620_0.5–2.5min_, continued with [^18^F]FDG-PET and β-amyloid-PET_0–10min_; Reader 3 started with β-amyloid-PET_0–10min_, continued with [^18^F]PI-2620_0.5–2.5min_ and finished with [^18^F]FDG-PET. Overall, n=217 reads were carried out by each reader for 3/4R-Tauopathies (n=77; [^18^F]PI-2620_0.5–2.5min_: n=38, β-amyloid-PET_0–10min_: n=24 and [^18^F]FDG-PET: n=15) and 4R-Tauopathies (n=140; [^18^F]PI-2620_0.5–2.5min_: n=68, β-amyloid-PET_0–10min_: n=36 and [^18^F]FDG-PET: n=36). Due to artifacts that occurred during normalization to the 3D-SSP (e.g., dilatation of ventricular system, pronounced atrophy, post-resection), one [^18^F]FDG-PET scan was excluded from the 3/4RT cohort for further analysis. In the 4RT group, two [^18^F]FDG-PET scans, two early-phase β-amyloid-PET scans, and two [^18^F]PI-2620 scans were excluded.

*AI Algorithm for volumetric MRI*

For quantitative analysis of brain MRI, the commercially available AI tool "mdbrain", version 3.3.0 (mediaire GmbH, Berlin) was used. The system leverages a custom deep learning segmentation model based on the U-Net architecture [3] to perform a highly accurate hemisphere- and region-specific (e.g., lobes) rapid brain volumetry in clinical routine settings, which was trained on a heterogeneous dataset of 3D T1w images (n = 1,851, balanced male/female). Augmentation techniques (augmentation of contrast, resolution, rotation, elastic deformation) have been used to maximize the model’s applicability in daily routine. The volumes of 18 brain regions including the hippocampus are determined and percentiles are derived by comparison to a cohort of healthy individuals (n=3,179, balanced m/f, age range 18 to 92), while accounting for age, sex, and total intracranial volume.

**Supplementary Results**

*Delivery of [^18^F]PI-2620 depicts neuronal damage in patients with tauopathies (N)*

First, we investigated which kinetic parameter best reflects neuronal damage. Among the three parameters tested, R1 demonstrated the highest predictive value (AUC=0.8978), although the differences did not reach statistical significance when compared to K2a (AUC=0.7828, p=0.054) and DVR (AUC=0.8509, p=0.74), using the binary consensus-based visual read of early-phase [^18^F]PI-2620 3D-SSP PET images as the reference. In addition, reduced R1 values showed the strongest association with averaged visual read severity (r=-0.41, p<0.0001), outperforming K2a (r=-0.36, p=0.0002) and DVR (r=0.10, p=0.33). These results are consistent with previous studies that have successfully implemented R1 as a surrogate marker of neuronal injury [4]. Based on this evidence, and given its superior association with visual read severity, R1 was selected as the most suitable kinetic parameter to represent the N component in our proposed A/T/N framework. Next, we performed an intermodal correlation analysis between [^18^F]PI-2620_R1_, [^18^F]FDG-PET and early-phase β-amyloid-PET in our combined tauopathy cohort. We observed a significant correlation between [^18^F]PI-2620_R1_ and [^18^F]FDG-PET in the frontal cortex (R=0.24; P=0.017), the temporal cortex (R=0.26; P=0.0089), the parietal cortex (R=0.53; P<0.0001), the occipital cortex (R=0.31, P=0.0019), and the basal ganglia (R=0.24; P=0.0018). The insular cortex, although indicating a clear trend, did not fully reach the significance threshold of P<0.05 (R=0.19; P=0.065). Similarly, [^18^F]PI-2620_R1_ was significantly associated with early phase β-amyloid-PET in the frontal cortex (R=0.21; P=0.023), the temporal cortex (R=0.38, P<0.0001), the parietal cortex (R=0.56; P<0.0001), the insular cortex (R=0.32; P=0.0003), the occipital cortex (R=0.42; P<0.0001) and the basal ganglia (R=0.29; P=0.0011) (**Supplementary Figure 8**).

Additionally, in a subset of 4RT (n=43) and 3/4RT (n=22), volumetric MRI was acquired to obtain atrophy scores (in %) for both cortical hemispheres and hindbrain regions. Subcortical bilateral volumes were successfully obtained in 4RT (n=32) and 3/4RT (n=12), respectively (**Supplementary Figure 9**). When correlating [^18^F]PI-2620_R1_ with the degree of atrophy, a significant positive association was observed in all cortical regions of 3/4RT including the frontal cortex (R=0.57; P<0.0001), the temporal cortex (R=0.41; P=0.0051), the parietal cortex (R=0.64; P<0.0001) and the occipital cortex (R=0.49; P=0.0007). Perfusion deficits in the cortex of 4RT were significantly correlated with atrophy in the parietal lobe (R=0.23; P=0.03), while in the frontal lobe, only a positive trend was indicated (R=0.20; P=0.065). No significance, however, was attained in the temporal (R=0.07; P=0.50) and occipital cortex (R=0.07; P=0.51) (**Supplementary Figure 9A**). Considering the subcortical regions, [^18^F]PI-2620_R1_ was negatively correlated with atrophy in the globus pallidus (R=-0.29; P=0.018) of 4RT, while a moderate degree of correlation was found in the nucleus caudatus (R=0.27; P=0.03). The relative perfusion of the putamen of the same patient sample was borderline negatively associated with atrophy (R=-0.24; P=0.053) among both imaging modalities. In the 3/4RT group, only the putamen revealed a significant negative association among [^18^F]PI-2620_R1_ and the level of atrophy (R=-0.44; P=0.031) (**Supplementary Figure 9B**).

*High visual agreement among early-phase [^18^F]PI-2620 and β-amyloid-PET as well as [^18^F]FDG-PET (N)*

In terms of clinical translation of the proposed single session A/T/N paradigm, the assessment of tracer efflux will require computational tools, whereas tau-PET can be evaluated visually [5, 6]. Hence, we asked if neurodegeneration can also be obtained from early-phase tau-PET imaging by a visual read. Visual assessment was performed by three experienced readers evaluating global mean normalized 3D-SSP images of early-phase [^18^F]PI-2620_0.5–2.5min_, β-amyloid-PET_0–10min_ and [^18^F]FDG-PET (**Supplementary Figure 10A)**. Representative images of early-phase [^18^F]PI-2620_0.5–2.5min_, β-amyloid-PET_0–10min_ and [^18^F]FDG-PET 3D-SSP are visualized in **Supplementary Figure 10B**. First, we explored the tracer specific ratings among the readers using Fleiss kappa. The highest degree of congruency was attained for [^18^F]FDG-PET scans of 3/4RT (k=0.87; p<0.001), 4RT (k=0.83; p<0.001), and both cohorts combined (k=0.84; p<0.001). A high agreement was also found among the early-phase β-amyloid-PET reads of 3/4RT (k=0.75; p<0.001), 4RT (k=0.50; p<0.001), and both groups combined (k=0.59; p<0.001). Similarly, early-phase tau-PET reads indicated a strong congruency in 3/4RT (k=0.59; p<0.001), 4RT (k=0.60; p<0.001), and both groups (k=0.60; p<0.001). Next, we investigated the disease specific ratings among the different PET modalities using Cohen’s kappa. [^18^F]FDG-PET vs. early-phase β-amyloid-PET indicated the highest degree of agreement in the 3/4RT (k=1.0; p<0.001) followed by [^18^F]FDG-PET vs. early-phase tau-PET (k=0.59; p<0.001), and both early-phase reads (k=0.48; p<0.001). In patients with 4RT we found the strongest agreement between [^18^F]FDG-PET vs. early-phase tau-PET (k=0.74; p<0.001), followed by [^18^F]FDG-PET vs. early-phase β-amyloid-PET (k=0.67; p<0.001) and complemented by the couple of both early-phase PET modalities (k=0.54; p<0.001) (**Supplementary Figure 10C**). In summary, visual assessment of early-phase tau-PET imaging delivers a reliable and robust surrogate of neurodegeneration across primary and secondary tauopathies.

**Supplementary Figure 1**

**Supplementary Figure 1:** **Comparison of disease progression between 4R and 3/4R.** An unpaired, two-tailed t-test was used for all comparisons.

**Supplementary Figure 2**

**Supplementary Figure 2: Comparison of tau-PET DVR and K2a maps in individuals with DLB and amnestic mild cognitive impairment due to AD (AD-MCI).** (**A**) Two representative cases with probable DLB [7]: one β-amyloid- and tau-negative, the other β-amyloid- and tau-positive. (**B-C**) Six AD-MCI cases: four with visually negative tau-PET reads and two with low-level tau positivity. All images are presented as z-score maps normalized to the control cohort and overlaid on a standard MRI template in MNI space.

**Supplementary Figure 3**

**Supplementary Figure 3: Comparison of [^18^F]PI-2620_K2a_ in the CBS subpopulation.** An unpaired, two-tailed t-test was performed to compare β-amyloid-negative (n=12) and β-amyloid-positive (n=11) CBS patients in the predefined K2a-based discriminatory ROIs.

**Supplementary Figure 4**

**Supplementary Figure 4: Assessment of K2a and DVR in the PSP and CBS subgroups within the 4RT cohort.** (**A,C**) Group-averaged [^18^F]PI-2620 K2a and DVR z-score maps of β-amyloid negative PSP and CBS participants, displayed as axial overlays on a standard MRI template in MNI space. (**B, D**) ANOVA-based comparison of K2a and DVR across 24 predefined subregions of the Brainnetome atlas between PSP and CBS cases. Significance levels are indicated as: *p < 0.0332, **p < 0.0021, ***p < 0.0002, ****p < 0.0001.

**Supplementary Figure 5**

**Supplementary Figure 5: Relationship between K2a and Centiloid units.** Correlation plots visualize association between [^18^F]PI-2620 K2a and Centiloid units in the 10% most discriminating K2a-based regions and the CenTauR Meta Temporal ROI in both study cohorts.

**Supplementary Figure 6**

**Supplementary Figure 6: Groupwise comparison of p-tau-181, t-tau and NfL.** P-tau-181 and t-tau levels were compared between 4RT (n=56) and 3/4RT (n=39), while NfL was analyzed in a subset of both groups (4RT: n=48, 3/4RT: n=11). An unpaired, two-tailed t-test was used for all comparisons.

**Supplementary Figure 7**

**Supplementary Figure 7:** **Evaluation of [¹⁸F]PI-2620 kinetic parameters for the assessment of neurodegeneration.** (**A**) ROC curves illustrating the results of a logistic regression analysis using R1, K2a, and DVR to predict neuronal damage. Neuronal damage was defined as a binary outcome based on consensus visual assessment of [^18^F]PI-2620_0.5–2.5min._ (**B–D**) Partial correlation analyses accounting for the cohort as a covariate, examining the relationship between R1, K2a, and DVR and the average severity of neurodegeneration as assessed by the three independent readers.

**Supplementary Figure 8**

**Supplementary Figure 8: Relationship between the relative perfusion (R1) of [^18^F]PI-2620 and early-phase β-Amyloid-PET, [^18^F]FDG-PET.** Whole brain scaled SUVR of early phase β-amyloid-PET (blue dots) and [^18^F]FDG-PET (red dots) are correlated with [^18^F]PI-2620_R1_ in cortical and subcortical regions of 4RT and 3/4RT.

**Supplementary Figure 9**

**Supplementary Figure 9: Correlation charts of [^18^F]PI-2620_R1_ and atrophy in distinct brain regions.** (**A-B**) Correlation plots depict the agreement of [^18^F]PI-2620_R1_ and volumetric MRI-acquired atrophy in cortical (**A**) and subcortical (**B**) brain regions of 4RT (green dots) and 3/4RT (purple dots) participants.

**Supplementary** **Figure 10**

**Supplementary** **Figure 10: Visual evaluation of early-phase [^18^F]PI-2620 PET for detection of neurodegeneration.** (**A**) Heatmaps of visual classification performed by dichotomous rating of [^18^F]PI-2620_0.5–2.5min_, β-amyloid-PET_0–10min_ and [^18^F]FDG-PET scans by three independent readers, who defined positivity (N+) or negativity (N-) for neuronal injury. (**B**) Representative 3D-SSP images (z-score maps) of congruent [^18^F]PI-2620_0.5–2.5min_, β-amyloid-PET_0–10min_ and [^18^F]FDG-PET scans. **B1**: 4RT rated *N+* by all three readers; **B2**: 3/4RT rated *N+* by all three readers; **B3**: 4RT rated *N+* by all three readers only in [^18^F]PI-2620_0.5–2.5min;_ PET; **B4**: 4RT rated *N-* by all three readers; **B5**: 4RT rated *N+* only in [^18^F]PI-2620_0.5–2.5min_ and [^18^F]FDG-PET; **B6**: 3/4RT rated *N+* by all three readers only in [^18^F]PI-2620_0.5–2.5min._ (**C**) Degree of agreement between the three readers for all three imaging modalities and both study cohorts depicted by Fleiss kappa (κ). Degree of congruency between two different scan modalities for both study cohorts was measured using Cohens Kappa (κ).

**Supplementary Figure 11**

**Supplementary Figure 11: Relationship between the relative perfusion (R1) of [^18^F]PI-2620 and total tau protein.** Green and purple scatterplots depict negative agreement among whole brain [^18^F]PI-2620_R1_ and total tau protein in the cerebrospinal fluid of both cohorts.

**Supplementary References**

1.	Brendel, M., et al., *Aβ status assessment in a hypothetical scenario prior to treatment with disease-modifying therapies: Evidence from 10-year real-world experience at university memory clinics.* Alzheimer's & Dementia: Diagnosis, Assessment & Disease Monitoring, 2024. **16**(4): p. e70031.

2.	Minoshima, S., et al., *A diagnostic approach in Alzheimer's disease using three-dimensional stereotactic surface projections of fluorine-18-FDG PET.* J Nucl Med, 1995. **36**(7): p. 1238-48.

3.	Ronneberger, O., P. Fischer, and T. Brox. *U-Net: Convolutional Networks for Biomedical Image Segmentation*. in *Medical Image Computing and Computer-Assisted Intervention – MICCAI 2015*. 2015. Cham: Springer International Publishing.

4.	Beyer, L., et al., *Early-phase [18F]PI-2620 tau-PET imaging as a surrogate marker of neuronal injury.* European Journal of Nuclear Medicine and Molecular Imaging, 2020. **47**(12): p. 2911-2922.

5.	Brendel, M., et al., *Assessment of 18F-PI-2620 as a Biomarker in Progressive Supranuclear Palsy.* JAMA Neurology, 2020. **77**(11): p. 1408-1419.

6.	Fleisher, A.S., et al., *Positron Emission Tomography Imaging With [18F]flortaucipir and Postmortem Assessment of Alzheimer Disease Neuropathologic Changes.* JAMA Neurol, 2020. **77**(7): p. 829-839.

7.	McKeith, I.G., et al., *Diagnosis and management of dementia with Lewy bodies: Fourth consensus report of the DLB Consortium.* Neurology, 2017. **89**(1): p. 88-100.
